# The Effect of Physical Activity on University Students’ Psychological Resilience: The Mediating Role of Self-Control

**DOI:** 10.21315/mjms-03-2026-197

**Published:** 2026-08-31

**Authors:** Xiawei Wang, Zhutang Liu, Yee Cheng Kueh, Haiyan Zhang, Yali Zhou, Garry Kuan

**Affiliations:** 1 Exercise and Sports Science Programme, School of Health Sciences, Health Campus, Universiti Sains Malaysia, 16150 Kubang Kerian, Kelantan, Malaysia; 2 Biostatistics and Research Methodology Unit, School of Medical Sciences, Health Campus, Universiti Sains Malaysia, 16150 Kubang Kerian, Kelantan, Malaysia; 3 Institute of Physical Education, Guangzhou Huali College, 511325 Guangzhou, China

**Keywords:** physical activity, psychological resilience, self-control, university students

## Abstract

**Background:**

University students frequently encounter academic, interpersonal and financial pressures, which may increase their vulnerability to psychological distress. Physical activity has been identified as a protective factor for mental health, potentially enhancing psychological resilience through improved self-control. This study aimed to examine the relationship between physical activity and psychological resilience among university students and to investigate the mediating role of self-control.

**Methods:**

A cross-sectional survey was conducted with 726 university students (mean age = 19.2 years, SD = 0.92). The participants completed the Physical Activity Rating Scale-3, the Self-Control Scale, and the Connor–Davidson Resilience Scale. Descriptive statistics, correlation analyses, regression analyses, and mediation analysis were performed using SPSS 28.0 and PROCESS 4.0.

**Results:**

Significant differences in self-control (*F* = 29.67, *P* < 0.001) and psychological resilience (*F* = 21.95, *P* < 0.001) were observed across physical activity levels. Physical activity positively predicted self-control (*β* = 0.269, *P* < 0.001) and psychological resilience (*β* = 0.251, *P* < 0.001). Self-control was a significant predictor of psychological resilience (*β* = 0.511, *P* < 0.001). Mediation analysis indicated that self-control partially mediated the relationship between physical activity and psychological resilience, accounting for 51.46% of the total effect.

**Conclusion:**

Physical activity enhances psychological resilience directly and indirectly through the improvement of self-control. These findings highlight the potential value of promoting regular physical activity as an effective strategy for strengthening psychological resilience and improving mental well-being among university students.

## Introduction

The university period is a critical phase in the development of mental health and healthy behaviours. In recent years, psychological disorders such as depression, anxiety, and eating disorders among university students have become increasingly concerning (1, 2). Globally, most students enter universities immediately after high school. This transition from a dependent high school life to an independent university setting brings significant challenges. Many young individuals face difficulties in interpersonal relationships and social adaptation, which often exceed their coping capacities. Moreover, university students are now presented with various uncertainties originating from socio-economic alterations following the COVID-19 pandemic, including unemployment, increasing expenses, and reduced immunity, which have introduced further uncertainties. These variables may negatively impact their energy levels, concentration, cognitive abilities, and overall mood (3–5). Under these complex stressors, it is essential to establish effective psychological regulatory mechanisms. Self-control and psychological resilience are pivotal in enabling students to manage immediate challenges and prolonged stress (6).

Psychological resilience, as a core psychological trait, refers to the ability of individuals to recover, adapt, and maintain psychological functioning when facing prolonged stress, adversity, or significant life changes (7, 8). It plays a crucial role among university students by aiding emotional regulation, cognitive reappraisal, and active coping (9). Research has shown that students with high resilience tend to adopt more positive emotional management strategies, effectively preventing the accumulation of negative emotions, and exhibiting greater adaptability and academic persistence (10). Studies on Chinese medical students reveal that psychological resilience can mediate the impact of negative life events on mental health. This suggests that strengthening resilience may promote better adjustment during students’ college or university period (11). Moreover, resilience enhances optimistic attitudes and the utilisation of social support networks, helping students alleviate feelings of isolation and improve their mental health (8, 12). However, the underlying processes linking psychological resilience in university students remain complex and warrant further exploration.

Self-control is a critical factor influencing psychological resilience. It is defined as the ability to manage emotions, inhibit impulsive behaviours and maintain goal-directed actions under stress (13, 14). Studies have demonstrated that self-control directly fosters the development of psychological resilience through cognitive adjustment, emotional regulation and behavioural inhibition (10, 15). For instance, individuals with higher self-control are better equipped to manage negative emotions, suppress impulsive actions and focus on long-term goals, thus establishing a solid foundation for resilience in challenging circumstances (16). University students with higher self-control are better equipped to maintain focus, resist distractions and excel academically and personally (10, 17). Additionally, they are more likely to maintain healthy lifestyles by establishing regular routines, following balanced diets, and engaging in physical activity. They also tend to avoid risky behaviours, such as excessive alcohol consumption and smoking (18). It may also serve as a key psychological mechanism linking behavioural factors and mental health outcomes.

Physical activity, characterised by its goal-oriented, challenging and collaborative nature, supports university students’ mental health and behavioural regulation while significantly enhancing self-control and psychological resilience (19, 20). During training and competition, university students must resist external distractions, overcome physical fatigue and manage their emotions. This ability to suppress short-term temptations and maintain focus on their goals significantly enhances their self-control levels (21). Challenging situations in sports, such as setbacks during competitions and high-intensity training, encourage individuals to adapt to stress through cognitive restructuring and emotional regulation strategies (22). Moreover, participating in team-based sports fosters experiences of overcoming setbacks and sharing achievements with teammates, which not only enhances individual self-efficacy but also provides a social support network to help them cope with long-term stress (23).

Despite existing evidence highlighting the positive effects of physical activity on self-control and psychological resilience, the specific mechanisms underlying these relationships remain insufficiently explored (23, 24). In particular, the mediating role of self-control in linking physical activity to psychological resilience remains underexplored. Cultural differences may significantly influence the relationships among physical activity, self-control and resilience (10, 25). Against the backdrop of intense academic pressure and rapid social transitions in China, investigating the mechanisms by which physical activity contributes to university students’ mental health is of great practical significance.

This study aimed to examine the relationship between physical activity and psychological resilience among Chinese university students and to explore the mediating role of self-control in this relationship. Based on the existing literature, the following hypotheses are proposed: i) Physical activity is positively associated with psychological resilience; ii) physical activity is positively associated with self-control; iii) self-control is positively associated with psychological resilience; and iv) self-control mediates the relationship between physical activity and psychological resilience. By testing these hypotheses, this study seeks to address gaps in the existing literature and provide theoretical and practical guidance for mental health interventions targeting university students.

## Methods

### Study Design and Procedure

The study was granted ethical approval by the Human Research Ethics Committee of Universiti Sains Malaysia under the code USM/JEPeM/KK/23040346. This study employed a cross-sectional design, collecting data at a single time point through convenience sampling. The study specifically targeted first- and second-year students from five universities in Henan Province, China. This decision was based on the fact that students in these year groups are required to participate in physical education courses at these universities. Including students actively engaged in physical education courses aligns with the study’s objectives to examine the role of physical activity in enhancing psychological resilience and self-control.

Data were collected using a web-based questionnaire administered through the Sojump platform. The instruments used in this study were formally authorised by their respective developers or copyright holders. Before participation, all students were required to read and agree to an electronic informed consent form displayed on the questionnaire homepage. The consent form clearly outlined the purpose of the study, the voluntary nature of participation, data confidentiality, and the study’s inclusion and exclusion criteria. Only students who provided informed consent could proceed to complete the questionnaire. The questionnaire link was distributed with the assistance of university administrators to ensure it reached eligible participants. To maintain data quality, incomplete questionnaires were automatically excluded by the survey platform. Participants completed the questionnaire during their break times, and no monetary or material incentives were provided for participation.

### Participants

A total of 750 questionnaires were distributed, and 726 valid questionnaires were retained for analysis after excluding incomplete responses. The mean age of the participants was 19.2 years (SD = 0.92), with 50.7% males and 49.3% females. The sample consisted of first- and second-year university students who were actively enrolled in physical education courses at five universities. This sampling strategy was adopted because physical education courses are compulsory for first- and second-year students in Chinese universities, whereas senior students are not required to take these courses and often have irregular campus attendance due to internships or graduation-related activities. Eligibility criteria included: i) being a full-time university student; ii) being enrolled in physical education courses; and iii) implied informed consent obtained through completion of the online questionnaire, where the study purpose and participation information were presented on the first page. Students who submitted incomplete questionnaires were excluded from the final analysis.

Although no a priori sample size calculation was conducted due to the cross-sectional design, the final sample size (*n* = 726) is considered sufficient based on methodological recommendations for regression and structural equation modelling (26). This sample size exceeds commonly suggested minimum requirements, ensuring adequate statistical power and stable parameter estimation. To ensure data quality, incomplete responses were automatically excluded by the survey platform, and students who declined to participate were not included. The descriptive statistics are presented in Table 1.

### Measures

#### Physical Activity Rating Scale-3

The Physical Activity Rating Scale (PARS-3), developed by Kimio Hashimoto and adapted by Deqing Liang, was used to measure the physical activity of university students (27, 28). This scale is a widely used physical activity measurement tool due to its simplicity and convenience, and its good reliability and validity (29, 30). It evaluates three dimensions of physical activity: intensity, time, and frequency, and uses a Likert 5-point scale. The physical activity score can be calculated as “physical activity intensity × time × frequency” and its assessment criteria are ≤ 19 as low volume exercise, 20 to 42 as moderate volume exercise, and ≥ 43 as high volume exercise. The internal consistency of the scale in this study was 0.701.

#### Self-Control Scale

The Self-Control Scale was used to measure the self-control of university students. This scale was developed by Tangney et al. (13) and adapted to Chinese by Tan and Guo (31). This scale is widely applied in Chinese populations and consists of 19 questions on five dimensions: impulse control, healthy habits, academic performance, focused work, and resistance to temptation. A Likert 5-point scale was used, with 11 questions scored in reverse, and with higher scores indicating greater self-control. The internal consistency of this scale in this study was 0.872.

#### The Connor–Davidson Resilience Scale

The Connor–Davidson Resilience Scale (CD-RISC), developed by Connor and Davidson, is a commonly used scale to measure psychological resilience (32). In this study, the CD-RISC scale was adapted to Chinese by Yu and Zhang (33). The 25-item scale consists of three dimensions: toughness, strength, and optimism. The scale is scored on a 5-point scale, ranging from 0 (never) to 4 (always). Previous studies have demonstrated its suitability for Chinese populations, with strong reliability and validity (34, 35). The internal consistency of the scale in this study was 0.956.

### Statistical Analysis

The SPSS 28.0 and Process 4.0 software were used to statistically analyse the datasets. To avoid systematic bias due to factors such as data collection methods, measurement tools, measurement environments, or the respondents themselves, all data were first subjected to a common method bias test. The Harman one-way test was used to test the collected data for common method bias, and all measurement items were factor analysed. If the variance explained by the first principal component was less than 50%, the data were considered free of common method bias and deemed reliable and valid (36).

The internal consistency of the individual scales was also verified using Cronbach’s coefficient, which ranges from 0 to 1, with higher values indicating better internal consistency; coefficients greater than 0.7 are considered acceptable (37). Descriptive analyses were used to represent the demographic characteristics of the participants, with correlation and regression analyses to examine the relationship between physical activity, self-control, and psychological resilience (38, 39). Mediation effects were tested using Hayes’ Process macro (model 4), with significance determined by bootstrap estimates of regression coefficients (*β*), *R*^2^ values, 95% confidence intervals (CIs), and *p*-values (40). A mediation effect is considered significant if the 95% CI for the indirect effect does not include zero. Additionally, a *P*-value of less than 0.05 indicates statistical significance, and higher *R*^2^ values suggest greater explanatory power of the model (41).

## Results

### Control and Testing of Common Method Bias

The study was controlled for common method bias procedurally through measures such as anonymous measurement and partial item reversal, and the Harman one-way test was used to test the collected data for common method bias (36). The results showed that the unrotated exploratory factor analysis results extracted three factors with a characteristic root > 1 and a maximum factor variance explained as 32.55% (< 50%). It can be concluded that the data from this study do not suffer from high common method bias.

### Preliminary Correlation Analysis of Physical Activity, Self-Control, and Psychological Resilience

Significant positive correlations between i) physical activity and self-control (*r* = 0.269; *P* < 0.001); ii) physical activity and psychological resilience (*r* = 0.251; *P* < 0.001); and iii) psychological resilience and self-control (*r* = 0.511; *P* < 0.001). These findings are presented in Table 2.

### The Effect of Physical Activity on Self-Control and Psychological Resilience

Based on the physical activity score, calculated as “physical activity intensity × time × frequency,” the total score of physical activity was categorised into three levels: low (≤ 19), moderate (20 to 42), and high (≥ 43) volume physical activity. These classifications were used to perform the analysis of variance (ANOVA) to examine the effects of physical activity volume on self-control and psychological resilience among university students.

The means of self-control and psychological resilience were tested to observe more clearly and accurately the changes in the effects of physical activity volume on self-control and psychological resilience among university students. The results in Table 3 show that there were significant differences in self-control (*F* = 29.67; *P* < 0.001) and psychological resilience (*F* = 21.95; *P* < 0.001) between students of different physical activity volumes. The self-control and psychological resilience of the moderate volume and the high volume exercise groups were significantly higher than those of the low volume exercise group.

Regression analyses were conducted to examine the relationship between the variables, and the results in Table 4 indicate that physical activity positively predicted self-control (*β* = 0.269; *P* < 0.001; *R*^2^ = 0.072), and physical activity was positively associated with psychological resilience (*β* = 0.251; *P* < 0.001; *R*^2^ = 0.063). Besides that, self-control positively predicted psychological resilience (*β* = 0.511; *P* < 0.001; *R*^2^ = 0.261), and when physical activity and self-control were entered into the regression analysis at the same time, physical activity (*β* = 0.123; *P* < 0.001) and self-control (*β* = 0.478; *P* < 0.001) positively predicted psychological resilience (*R*^2^ = 0.275).

### The Mediating Role of Self-Control Between Physical Activity and Psychological Resilience

The results in Table 5 indicate that the confidence interval for the direct effect of physical activity on psychological resilience does not include 0 (95% CI: 0.04, 0.127), indicating a significant direct effect of physical activity on psychological resilience. Similarly, the confidence interval for self-control (95% CI: 0.062, 0.116) also does not include 0, indicating a significant indirect effect of self-control between physical activity and psychological resilience, playing a partial mediating role.

## Discussion

This study demonstrated that physical activity exerted direct and indirect effects on psychological resilience, with self-control serving as a significant partial mediator. Specifically, higher levels of physical activity were associated with greater psychological resilience and improved self-regulatory capacity. In addition, self-control partially explained the relationship between physical activity and psychological resilience, suggesting that students who are more physically active tend to develop stronger self-control abilities, which, in turn, contribute to better psychological adaptation. These findings highlight the important role of physical activity in promoting positive psychological functioning and resilience in university students.

Correlation analysis indicated significant positive relationships among physical activity, self-control, and psychological resilience, with coefficients ranging from 0.269 to 0.511. These results indicate that higher levels of physical activity are associated with stronger self-regulatory ability and greater psychological resilience among university students. In addition, the results of the ANOVA indicated significant differences in psychological resilience across different levels of physical activity (*F* = 21.95; *P* < 0.001). Students in the high volume physical activity group had significantly higher psychological resilience scores compared to those in the low volume group, highlighting the substantial benefits of engaging in higher levels of physical activity on mental health. These findings support the potential role of physical activity in enhancing psychological resilience.

The study further highlighted the importance of the duration, intensity and frequency of physical activity in shaping psychological resilience. Consistent with previous research, regular moderate-to-high-intensity exercise (e.g., three or more sessions per week lasting at least 30 min) was found to significantly alleviate psychological stress and enhance emotional regulation (42, 43). In line with these findings, the present study found that students with higher levels of physical activity reported significantly greater self-control and psychological resilience compared to those with lower levels of physical activity. Exercise has been shown to increase endorphin secretion and reduce cortisol levels, which contribute to improved mood states and reduced stress responses (44). These biological changes may help individuals better cope with daily stressors and maintain emotional stability, thereby strengthening psychological resilience. Moreover, team sports, characterised by cooperation and competition, may further boost students’ confidence and sense of social support, which are critical for enhancing psychological resilience (23, 45).

The mediating role of self-control in the relationship between physical activity and psychological resilience was particularly significant. Model testing indicated that the mediating effect of self-control accounted for 51.46% of the total effect, underscoring its critical role. Specifically, physical activity enhances self-control, enabling students to better manage emotions, suppress impulses and maintain goal-oriented behaviours when facing academic pressure, social challenges or personal adversities (13, 19). In turn, self-control directly predicted psychological resilience, reaffirming its central role in improving adaptability and emotional stability. These findings support the indirect effect of physical activity on psychological resilience through self-control. Consistent with previous studies (21, 46), this study underscores the potential of physical activity to indirectly foster the development of psychological resilience through cultivating self-discipline and goal perseverance.

The findings of this study align with previous research, demonstrating significant positive relationships among physical activity, self-control and psychological resilience, as well as between self-control and psychological resilience (18, 21). Physical activity promotes the production and release of endorphins while reducing levels of stress hormones such as adrenaline and cortisol. This process enhances positive emotional experiences, facilitates emotional expression and reduces negative emotions and stress, thereby helping students better cope with psychological pressure. Regular participation in physical activity may contribute to the development of psychological resilience by alleviating fatigue and anxiety and promoting a stable emotional state. Additionally, physical activity may help students realise their potential, boost self-confidence, overcome inertia, strengthen self-control and build perseverance in facing challenges, thereby ultimately enhancing psychological resilience (47, 48).

### Limitations

This study has several limitations that should be acknowledged. First, the cross-sectional design of this study restricts the ability to infer causality, as it only reveals associations between variables. Future research should adopt longitudinal or experimental designs to further elucidate the causal pathways among these variables and deepen the understanding of their mechanisms. Second, the reliance on self-reported data may introduce subjective bias or social desirability effects. For instance, participants might overestimate their physical activity frequency or levels of psychological resilience. Future studies could incorporate objective physiological indicators (e.g., heart rate variability or cortisol levels) and behavioural observations to enhance the objectivity and accuracy of the findings. Third, this study did not thoroughly examine the moderating effects of demographic variables such as gender and academic year, which could significantly influence the relationships between physical activity, self-control, and psychological resilience. Specifically, males and females may differ in their patterns of physical activity participation, frequency, and psychological characteristics. Future studies should conduct stratified analyses to explore potential group differences and provide tailored intervention strategies for different populations. Finally, this study did not explore the specific effects of different types of physical activity, such as team-based versus individual sports. These activity types may influence self-control and psychological resilience through distinct mechanisms. Namely, team sports may enhance social support and collaboration skills, whereas individual sports may focus more on self-discipline and emotional regulation. Future research could further compare the effects of these activity types to provide theoretical guidance for designing targeted psychological interventions.

## Conclusion

This study aimed to examine the relationship between physical activity and psychological resilience among university students, and to further explore the mediating role of self-control in this relationship. This study provides evidence that regular physical activity contributes to enhanced psychological resilience in university students both directly and indirectly through improvements in self-control. These findings underscore the dual pathway through which physical activity contributes to psychological resilience, highlighting its direct benefits and its role in fostering self-control as a key psychological mechanism. From a practical perspective, integrating self-regulation strategies such as goal setting, self-monitoring, and delay of gratification into physical education and psychological training programmes may further strengthen students’ self-control and resilience.

## Figures and Tables

**Table 1:** Description of the distribution of sample characteristics (*n* = 726).

	Characteristics	*n*	Percentage
Gender	Male	368	50.7
	Female	358	49.3

Grade	Freshman	364	50.1
	Sophomore	362	49.9

Hometown	City	251	34.6
	Country	475	65.4

**Table 2:** Descriptive statistics and correlation analysis for each variable.

Variable	Mean	SD	Physical activity	Self-control
Physical activity	25.43	24.13		
Self-control	72.27	8.48	0.269^a^	
Psychological resilience	87.55	16.37	0.251^a^	0.511^a^

SD = standard deviation;

a *P* < 0.001.

**Table 3:** Effects of different volumes of physical activity on self-control and psychological resilience (ANOVA).

Variable	Low volume^a^	Moderate volume^a^	High volume^a^	*F*	*P*-value
Self-control	70.46 (7.38)	72.93 (9.27)	76.39 (8.81)	29.67	< 0.001
Psychological resilience	84.26 (14.51)	89.85 (17.41)	93.79 (17.69)	21.95	< 0.001

a Mean (standard deviation); *F* = *F*-ratio (ANOVA)

**Table 4:** Outcomes of a mediated model test of self-control in physical activity and psychological resilience.

Result variables	Predictor variables	Fit index, *R*^2^	Coefficient significance		

			*t*-value	β	*P*-value
Self-control	Physical activity	0.072	7.504	0.269	< 0.001

Psychological resilience	Self-control	0.261	15.997	0.511	< 0.001

Psychological resilience	Physical activity	0.063	6.986	0.251	< 0.001

Psychological resilience	Physical activity	0.275	3.738	0.123	< 0.001
	Self-control		14.542	0.478	< 0.001

*R*^2^ = coefficient of determination; *t*-value = test statistic; *β* = standardised regression coefficient

**Table 5:** Mediating effects analysis of psychological resilience.

Effect	Variables	Estimate	BootstrapSE	95% CI	Estimate (%)
Total effect		0.171	0.024	0.123, 0.218	
Direct effect	Physical activity → Psychological resilience	0.083	0.022	0.04, 0.127	48.54
Indirect effect	Physical activity → Self-control → Psychological resilience	0.087	0.014	0.062, 0.116	51.46

Bootstrap SE = the standard error calculated using the bootstrap method; CI = confidence intervals.
